# Compressive Behavior, Microstructural Properties, and Freeze–Thaw Behavior of Tailing Recycled Aggregate Concrete with Waste Polypropylene Fiber Addition

**DOI:** 10.3390/ma14216712

**Published:** 2021-11-08

**Authors:** Fan Xu, Tao Li, Chenghua Li, Zhijun Li, Sheliang Wang, Nan Zhao

**Affiliations:** 1 School of Civil Engineering, Xi’an Technological University, Xi’an 710021, China; lzjsjh@aliyun.com; 2 Xi’an Key Laboratory of Civil Engineering Testing and Destruction Analysis on Military-Civil Dual Use Technology, Xi’an 710021, China; 3 College of Urban, Rural Planning and Architectural Engineering, Shangluo University, Shangluo 726000, China; litao623114@126.com; 4 School of Civil Engineering, Xi’an University of Architecture and Technology, Xi’an 710055, China; sheliangw@163.com; 5 School of Science, Xi’an University of Architecture and Technology, Xi’an 710055, China; m15809202074_1@163.com

**Keywords:** polypropylene fibers, tailing recycled aggregate concrete, stress-strain curve, compressive behavior, freeze–thaw, microstructure

## Abstract

To improve the high brittleness of recycled aggregate concrete containing iron ore tailings (TRAC), the feasibility of adding polypropylene fiber (PPF) to TRAC was studied by performing a compression stress–strain curve test, scanning electron microscope characterization, and a freeze–thaw cycle test. The results indicated that PPF had a beneficial impact on reducing the brittleness of TRAC. With the increase in PPF content, the peak strain increased, the elastic modulus decreased, and the peak stress and energy absorption capacity increased at first and then decreased. Furthermore, the microstructure investigation revealed that the interface friction between the PPF, aggregate, and cement matrix was the main source of energy dissipation. When the load acted on the concrete, the stress was dispersed to the fiber monofilaments, thus effectively enhancing the peak stress and peak strain of concrete and suppressing the generation and development of cracks in the concrete. In terms of freeze–thaw resistance, adding a small amount of PPF could reduce the negative effects of the freeze–thaw process on the cement matrix. On the premise of ensuring strength, the waste utilization should be as high as possible. Therefore, it was suggested that the content of PPF in fiber-reinforced tailings recycled aggregate concrete (TRAC-PP) should be 0.6%.

## 1. Introduction

With the advancement in industrialization and urbanization, infrastructural growth is increasing. Almost all countries in the globe suffer from major environmental problems such as depletion of natural resources and sustainable waste management [1]. The current traditional methods for waste concrete are landfills or littered in the open, which not only occupies large amounts of land but also causes the waste of resources. Many scholars at home and abroad have used crushed and graded waste concrete to replace natural coarse aggregate (NCA) to prepare recycled aggregate concrete (RAC) [2,3,4]. It was found that recycled coarse aggregate (RCA) had the characteristic features of lower tensile strength, weak interfacial zone (ITZ), and higher porosity compared with NCA [5,6,7], which limited the practical engineering applications of RAC. In addition to this, when RCA was incorporated in concrete at higher substitution levels, the freeze–thaw resistance of concrete was significantly reduced compared to natural aggregate concrete (NAC) [8,9].

Several studies have shown the enhancement of mechanical properties of RAC by the addition of iron ore tailings (IOTs) [10,11,12]. IOTs refer to the industrial solid waste associated with the process of iron ore mining. The stacked IOTs not only occupy vast land resources but also cause lasting environmental pollution. Furthermore, the main chemical compositions of IOTs are silicon and aluminum, which are similar to the component of cementitious materials [13]. This provides a prerequisite for the application of IOTs in the field of building materials. RAC prepared from IOTs as raw materials is called tailing recycled aggregate concrete (TRAC). Studying its mechanical and durability properties can provide a reference for the engineering application of TRAC.

However, our previous study found that in the stress–strain test, TRAC suddenly collapsed after peak stress. TRAC had the disadvantages of poor ductility and uncontrollable crack width after cracking, which limited its use in buildings [13]. It has been reported that a significant increase in the ductility of concrete can be achieved by polypropylene fibers (PPF) [14,15,16].

PPF has the characteristics of high strength, high impact resistance, and wear resistance, which are widely applied for producing polypropylene plastic bag and injection molding products [17,18,19,20,21]. Its traditional disposal methods for abandoned polypropylene waste are incineration or landfills, which pose serious risks to ecological balance and people’s health [22,23,24]. Currently, many scholars have carried out some studies on fiber-reinforced concrete. Xue et al. [14] studied the feasibility of adding PPF to cement–tailings matrix composites. It was found that the addition of PPF resulted in the failure mode change from brittle failure to ductile failure. Chen et al. [25] conducted the unconfined compressive strength test on the cemented paste backfill prepared by using PPF. The obtained test results showed that the addition of PPF to cemented paste backfill can enhance the stiffness, ductility, and stability. The research results of Malek et al. [26] indicated that the addition of waste PPF into high-performance concrete can substantially improve the compressive strength, splitting tensile strength and flexural strength. Combined with the results of mechanical property as analyzed above, it can be concluded that PPF can mitigate two opposing weakness: poor ductility in cement–tailings matrix composites and cracking resistance in plain concrete. However, it is vital to investigate that whether all the assumed hypotheses to design structures of cement–tailings matrix composites and plain concrete are also valid for fiber-reinforced tailings recycled aggregate concrete (TRAC-PP). Meanwhile, there is insufficient research on the mechanical properties of TRAC-PP. In mechanical terms, the greatest disadvantage of concrete materials is its vulnerability to cracking, which generally occurs at an early age in concrete. Based on the obtained results from our previous studies, cracking potentially reduces the lifetime of TRAC and causes serious durability and serviceability problems. Therefore, it was necessary to carry out an experimental program to investigate the mechanical properties of TRAC-PP. The mechanical properties included in this study are stress–strain curve, peak stress, peak strain, elastic modulus, and energy absorption capacity under compression. In addition, little information was available about microstructure changes, and the interaction between IOT and PPF was not mentioned.

For the promotion and application of fiber-reinforced concrete, durability properties must be considered, in addition to its mechanical properties [18,27,28]. Zhang et al. [29] has demonstrated that adding PPF to ordinary concrete helped to increase the resistance to the damage of freeze–thaw. Boz et al. [30] compared the freeze–thaw resistance of lime-stabilized clay reinforced with basalt fiber and PPF. Polypropylene fiber-reinforced lime-stabilized clay showed stronger resistance to freeze–thaw than basalt fiber-reinforced lime-stabilized clay.

However, empirical studies about the effect of PPF on the durability of TRAC are limited, especially under a freeze–thaw environment. Further studies are required to understand the effect of PPF on the mechanical properties and durability properties of TRAC. Therefore, in this paper, the compression stress–strain curve test was carried out to study the macroscopic mechanical properties. The microstructure was analyzed by scanning electron microscopy (SEM) to explain its macro-performance. Additionally, the freeze–thaw resistance properties were evaluated by weight loss rate, relative compressive strength changes, and relative dynamic modulus of elasticity (RDEM) changes. The obtained results would provide a basis for the application of TRAC-PP and provide a reference to the durability evaluation of concrete structures in service suffering from freeze–thaw cycles attack and durability design of concrete structures to be built.

## 2. Materials and Methods

### 2.1. Materials

**Figure 1:**
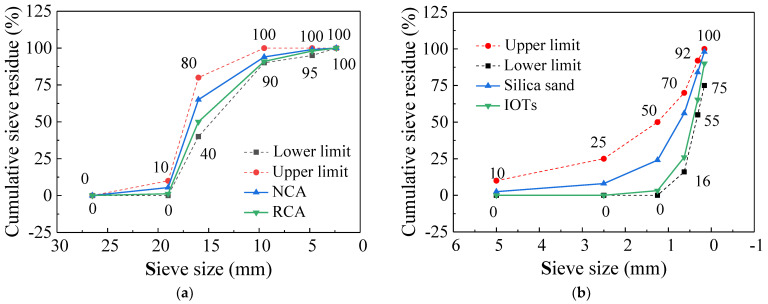
Particle gradation diagram. (**a**) Coarse aggregate. (**b**) Fine aggregate.

In this survey, RCA and NCA with particle sizes less than 20 mm were sourced from a quarry in Xi’an, Shaanxi. The brick content in RCA was 32%. The natural sand was Weihe River sand. IOTs were obtained from a tailings pond in Shaanxi Province. The particle gradation diagrams of fine aggregate and coarse aggregate are presented in Figure 1. Detailed physical information of fine aggregates and coarse aggregates is listed in Table 1. The elemental compositions of fine aggregates were detected by energy-dispersive spectroscopy and displayed in Table 2, indicating that silica sand and IOTs have the same chemical elements but different contents. Qinling Portland cement (PO 42.5) was adopted. The elemental compositions of cement are displayed in Table 2, and the main performance indicators of cement are shown in Table 3. Waste PPF was collected from discarded polypropylene woven bags, and the characteristics of waste PPF are provided in Table 4.

### 2.2. Mixture Proportion and Specimen Preparation

Five concrete mixtures were prepared with water–cement ratio of 0.4, as shown in Table 5. In our previous papers, RAC containing 30% IOTs exhibited the best mechanical properties [13,31,32,33]. Consequently, control concrete was prepared by adding 30% RCA instead of NCA and 30% IOTs instead of natural sand. To study the effects of PPF, PPF was added to the control concrete as 0.3%, 0.6%, 0.9%, and 1.2% by weight, respectively.

The mixtures were stirred vigorously with a horizontal double-shaft mixer. Firstly, coarse aggregates and fine aggregates were dry-mixed in the concrete mixer for 1 min. Then, PPF was added and mixed in the mixer at low speed for 1 min to evenly disperse the fibers. Subsequently, water and cement were added and stirred for 3 min. Finally, the mixtures were poured into a 100 mm × 100 mm × 300 mm mold for the freeze–thaw test and stress–strain curve test. Then, 10 s of external vibration was applied during concrete pouring to compact the mixtures. After curing at room temperature for 24 h, the mixtures were demolded. In accordance with the Chinese standards of GB/T 50081, the specimens were then cured at room temperature (24 ± 1 °C) and 90 ± 5% humidity until use.

### 2.3. Experimental Program

There were five different specimen groups considering the influences of PPF. Each group had 6 prismatic specimens (100 × 100 × 300 mm^3^) and 3 cubic specimens (100 × 100 × 100 mm^3^). Furthermore, each group had 3 sliced specimens, which were cut from the concrete after the cubic compressive strength test.

The uniaxial compressive stress–strain curves were determined on concrete by a WAW-1000 universal compression machine as specified in the GB/T50081-2002 standard test method.

**Figure 2:**
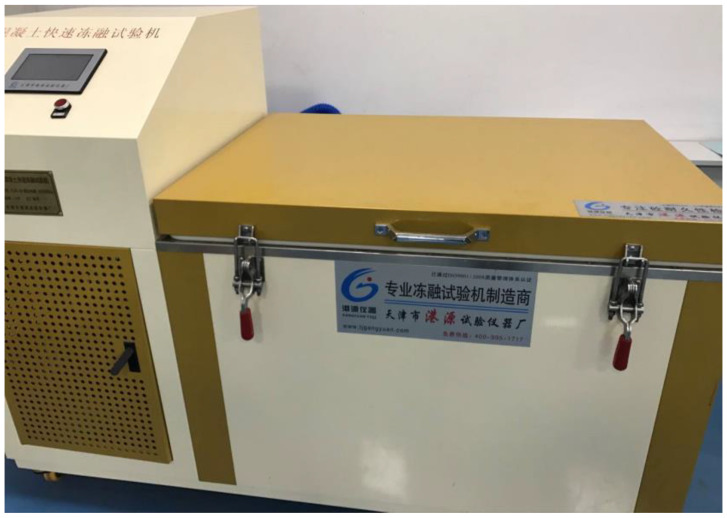
Automatic quick concrete freeze–thaw tester. Chinese in figure: Professional manufacturer of freeze-thaw testing machine, Tianjin Guangyuan Test Instrument Factory.

The freeze–thaw test was tested in the freeze–thaw test chamber according to the rapid-frozen method in the Chinese standard (GB/T 50082-2009). The test of freeze–thaw cycles is shown in Figure 2. The specimens were frozen for 2 h at −18.0 ± 1 °C and then thawed completely for 2 h at 5.0 ± 1 °C. The number of cycles was set to 25, 50, 75, and 100. After the specified number of cycles, the specimens were taken out of the freeze–thaw box and weighed with an electronic scale. Then, the nonmetal ultrasonic analyzer (ZBL-520, transducer frequency of 50 kHz) was used to measure the RDEM. After that, a compressive strength test was conducted by a WAW-1000 universal compression machine.

**Figure 3:**
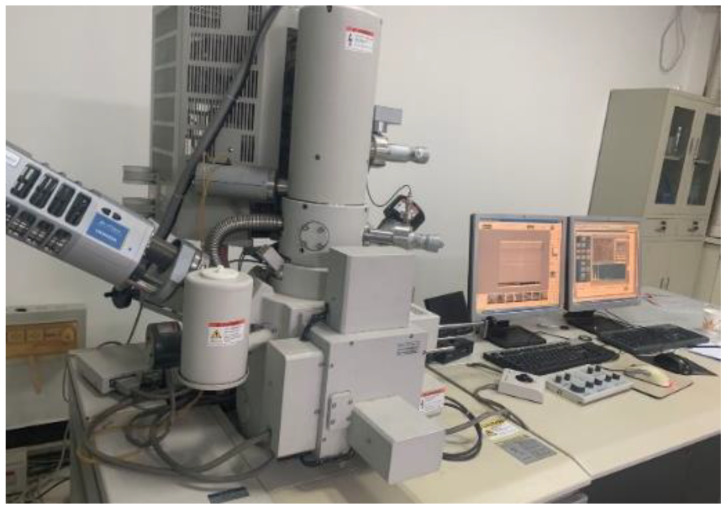
S-4800 scanning electron microscopy.

SEM (model Hitachi S-4800) was used to observe the hydration products and cracks of the surfaces on the concretes in accordance with the Chinese Standard GB/T 20307-2006, as shown in Figure 3. The specimens were immersed in anhydrous ethanol for 3 days to stop hydration. After vacuum drying, the specimens were placed in the SEM vacuum chamber for observation.

## 3. Result and Discussion

### 3.1. Stress–Strain Curve

**Figure 4:**
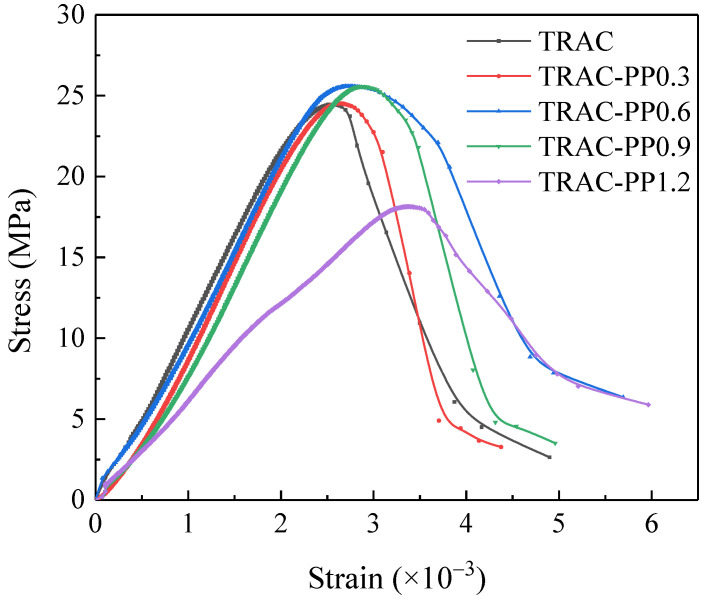
The compression stress-strain curves of specimens.

The stress–strain curves of concretes are presented in Figure 4. It can be seen in Figure 4 that the curves of each specimen followed a similar pattern that was composed of three main parts. The first part of the curve was almost linear, meaning that the theoretical behavior of fiber-reinforced tailings recycled aggregate concrete (TRAC-PP) followed Hooke’s law. The second part of the curve was the nonlinear part of the ascending branch. The slope of the nonlinear part was much less than the slope of the linear part. The descent stage of the curve was the third part. In the third part, the slope of TRAC curve is the largest, which indicated that TRAC failed in a more brittle manner. The slope of the descending part of the curve tended to flatten with the increase in PPF content, especially with 1.2% PPF content. This indicated that the plastic deformation capacity of concrete has been greatly promoted due to the bridging mechanism of PPF [34]. In addition, the transition area of the TRAC curve was narrow. Adding PPF widened the transition area of the stress–strain curves. This was a sign of improvement of ductility as well.

### 3.2. Parameters of Stress–Strain Curve

#### 3.2.1. Peak Stress

**Figure 5:**
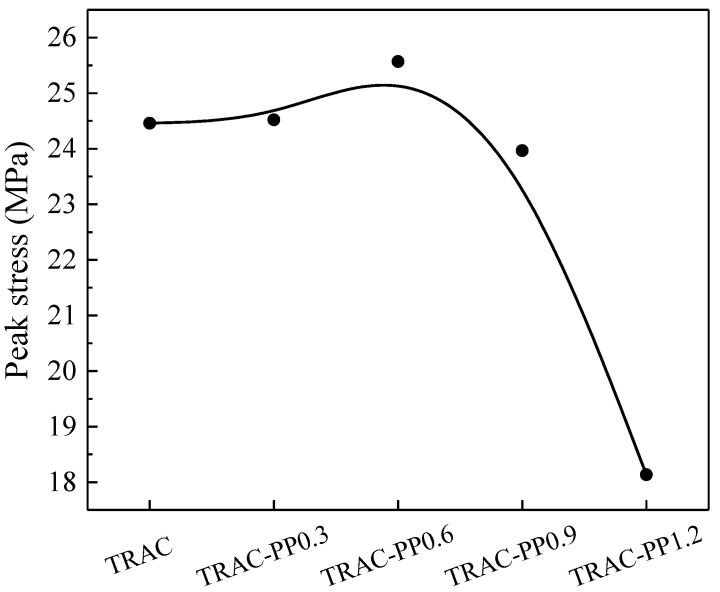
Effect of PPF content on the peak stress of TRAC-PP.

The peak stress of the tested specimens is shown in Figure 5. With the increase in PPF content, the peak stress of concrete increased first and then decreased. When the PPF content was 0.3%, there was no significant influence on peak stress. When the PPF content increased from 0 to 0.6%, the peak stress increased by 4.54%. Similar results were reported by Serbia [25], who reported that PPF, as a flexible fiber, can ensure good particle contact. They also reported that PPF’s high fracture energy led to a higher strength and better integrity of concretes to resist stress. However, when the added amount exceeded 0.6%, PPF was easily agglomerated and led to the formation of a weak area, which would therefore lead to reduced peak stress. Compared with TRAC control, the peak stress of TRAC-PP0.9 and TRAC-PP1.2 reduced by 2.02% and 25.86%. The reduction in peak stress of TRAC-PP0.9 and TRAC-PP1.2 can be attributed to the fact that too many fibers were intertwined, affecting the compactness of the concrete and weakening the bond between the aggregate and the matrix. Those points of view will be discussed further in Section 3.4.

#### 3.2.2. Peak Strain

**Figure 6:**
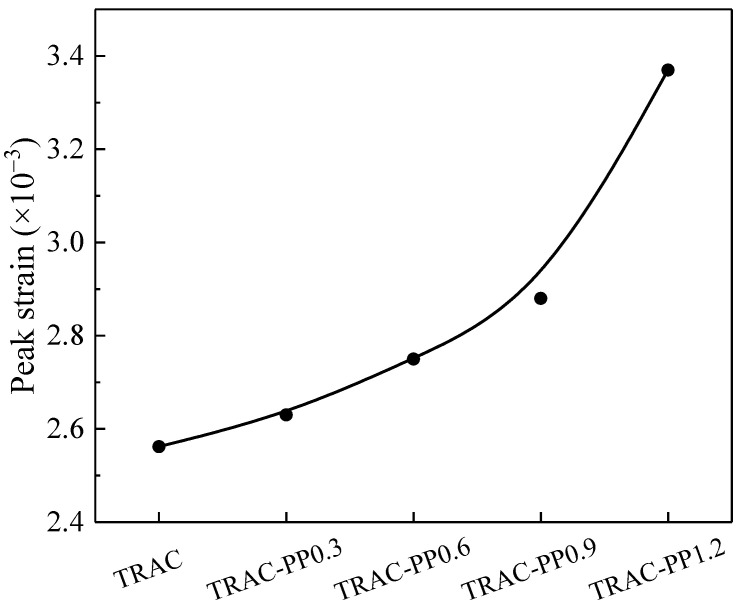
Effect of PPF content on the peak strain of TRAC-PP.

Figure 6 shows the variation curve of the peak strain with PPF addition. It was clear that as the PPF content increased, the peak strain of concrete increased and was greater than the control group (TRAC). Compared with the case in the absence of PPF (TRAC), the peak strains for TRAC-PP0.3, TRAC-PP0.6, TRAC-PP0.9, and TRAC-PP1.2 were increased by 2.7%, 7.4%, 12.5%, and 31.6%, respectively. It can also be observed that TRAC-PP1.2 showed the greatest improvement in deformation capacity. This illustrated again that the presence of PPF can improve the ductility of TRAC. The main reason for this increase in peak strain was that PPF can constrain further crack propagation at the crack initiation stage and withstand partial tensile stress from the surrounding matrix, which increased the additional strain capacity. The main reason for the increase in peak strain is that PPF can effectively inhibit the development of cracks and bear a part of the tensile stress in concrete, thus enhancing the ability of concrete to deform and expand its plastic zone. In addition, in TRAC-PP, PPF can hold the crack edges together during the crack initiation stage, retarding the further growth of crack and, consequently, preventing the generation of macro-cracks (for the detailed mechanism, see Section 3.4). As a result, the additional strain capacity of concrete was obtained.

#### 3.2.3. Elastic Modulus

**Figure 7:**
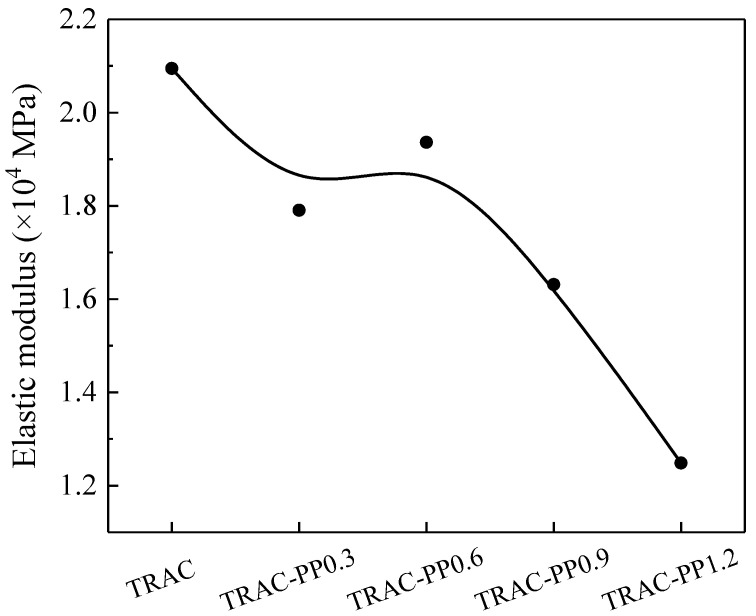
Effect of PPF content on the elastic modulus of TRAC-PP.

Figure 7 shows the elastic modulus of specimens at the curing age of 28 days. The elastic modulus of TRAC was 2.09 × 104 MPa, whereas the elastic modulus of TRAC-PP fell between 1.25 × 104 and 1.94 × 104 MPa. It can be seen that the all concretes containing PPF had lower elastic modulus than the TRAC control group. This occurred because the incorporation of PPF into TRAC introduced more internal defects, thus leading to a reduced capacity of a material to resist elastic deformation. When PPF was incorporated in TRAC, the ductility of the concrete was improved, and TRAC-PP exhibited more flexibility in the elastic segment of the stress–strain curve than TRAC; thus, the elastic modulus of TRAC-PP was relatively small. This could be also attributed to the low elastic modulus of PPF, making concrete more flexible. When concretes were under pressure, PPF occurred elastic deformation without breaking, which was also a process of energy dissipation.

#### 3.2.4. Energy Absorption Capacity

The energy absorption capacity was established by calculating the integrated area under the stress–strain curve and can be calculated using the following formula:

(1) $$E=\int_{0}^{\varepsilon }\sigma d\varepsilon$$

where *E* represents energy absorption capacity, σ represents the stress, and ε represents the strain.

The energy absorption capacity for TRAC and TRAC-PP specimens is reported in Table 6. The energy absorption capacity was largely affected by the orientation of PPF [35,36]: when more PPF ran perpendicular to the loading vector, the effect of fiber reinforcement on the energy absorption capacity was more visible. However, the fiber orientation distribution in the concrete was random. Consequently, the data of energy absorption fluctuated. Overall, except for TRAC-PP0.3, the energy absorption of all added PPF concretes was more than the energy absorption of TRAC. The addition of PPF to concrete reduced the stress intensity at the crack tip, which meant that further crack propagation required additional energy. When the amount of PPF added was 0.6%, the energy absorption capacity was the highest—it increased by 42.2% compared with no addition of PPF. Nevertheless, the energy absorption of concretes began to decrease when the content of PPF was higher than 0.6%. The energy consumed for crack generation would be much higher than the energy consumed for crack propagation. By bridging pre-existing cracks, PPF can change the expected crack propagation path and improve the energy absorption capacity.

### 3.3. The Non-Dimensional Stress–Strain Curves

To gain a better insight into the process of compression failure for TRAC-PP, the non-dimensional stress–strain curves with *σ*/*σ*_max_ as the ordinate and *ε*/*ε*_max_ as the abscissa was used in the present research to study the relationship between stress and deformation. Based on the research of Guo [37], the piecewise function method was applied to express the stress–strain curves:

(2) $$\begin{array}{l} y=\alpha x+(3-2\alpha )x^{2}+(\alpha -2)x^{3},0\leq x\leq 1\\ y=\frac{x}{\beta {\left(x-1\right)}^{2}+x},x\geq 1 \end{array}$$

where *x* = *ε*/*ε*_max_, in which *ε* is the strain and *ε*_max_ is the peak strain; *y* = *σ*/*σ*_max_, in which *σ* is the stress and *σ*_max_ is the peak stress; *α* and *β* are the shape parameters of ascending branches and descending branches of the curves.

**Figure 8:**
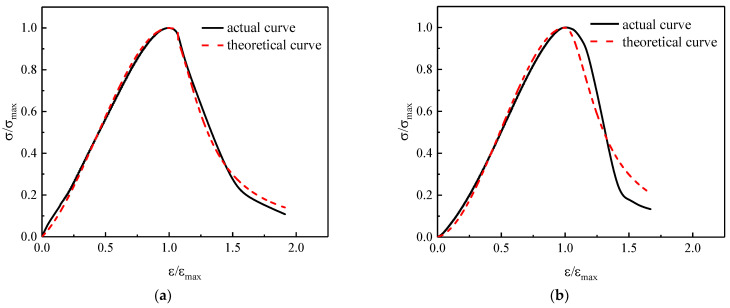

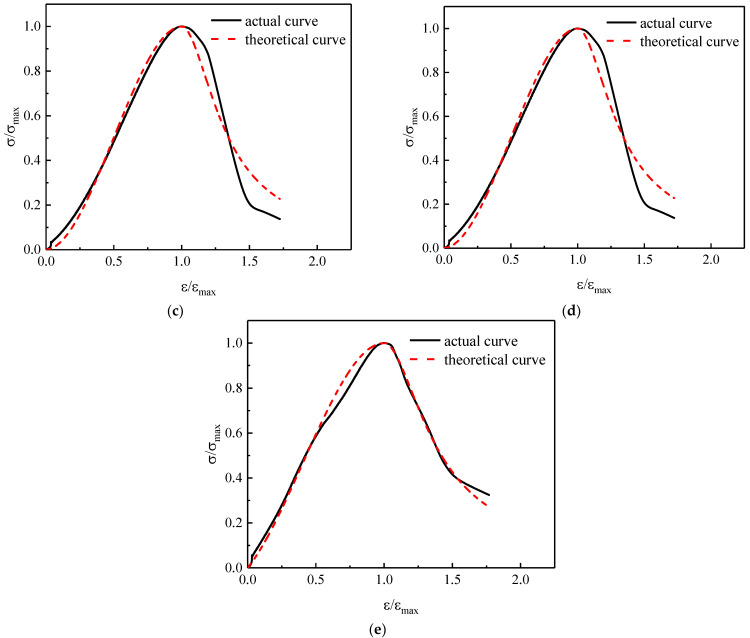
The comparison between fitting curves and test curves. (**a**) TRAC. (**b**) TRAC-PP0.3. (**c**) TRAC-PP0.6. (**d**) TRAC-PP0.9. (**e**) TRAC-PP1.2.

The results showed that the curvature of the ascending section curve decreased with the increase in *α*, while the curvature of the descending section curve increased with *β*. Figure 8 shows the comparison of fitting curves with test curves. The fitting curves are in good agreement with the test curves. The shape of the stress–strain curves was related to the energy absorption capacity of the concretes. That is to say, the values of *α* and *β* were affected by the energy absorption capacity of the concretes. The correlation between these two shape parameters and the energy absorption capacity was further analyzed, and the mathematic regression model was established, as shown in Equation (3).

(3) $$\begin{array}{l} \alpha =-317.758+14.552E-0.219E^{2}+0.001E^{3}\\ \beta =32.759-0.348E \end{array}$$

### 3.4. Microstructure

**Figure 9:**
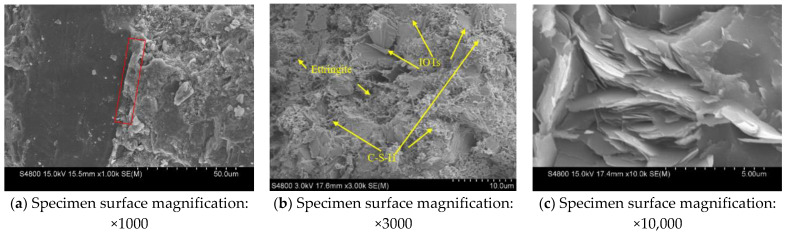

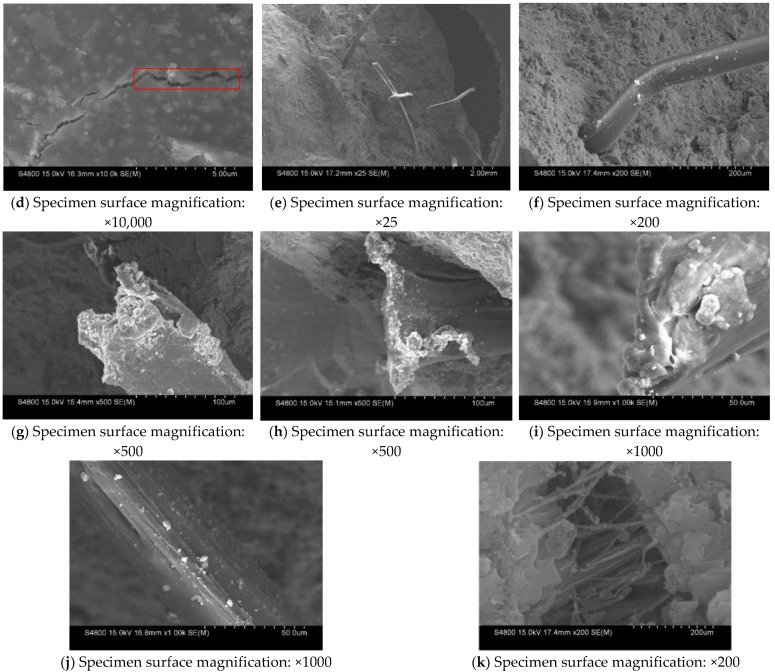
Microstructure of TRAC (**a**–**d**) and TRAC-PP0.6 (**e**–**k**).

To understand the relationship between the material composition and microstructure of TRAC and TRAC-PP series, SEM characterization was carried out, as shown in Figure 9.

Figure 9a–d show the SEM images of the specimen TRAC. The morphology of the interfacial transition zone (ITZ) between IOTs and the surrounding matrix is presented in Figure 9a, and the bonding appeared very dense. In Figure 9b, we observed a large amount of calcium silicate hydrate (C-S-H) gel, needle-like crystals (ettringite), and IOTs. IOTs had a certain pozzolanic activity and can react with calcium hydroxide to form C-S-H gel, which made the microstructure of the concrete denser, improving the strength of RAC [33,38]. This result was consistent with that of our previous study that showed that IOTs improved the compressive strength of RAC [13]. Similar results have been reported by Duan et al. [39,40,41]. From Figure 9c, it can be seen that there were a large number of monosulfate calcium sulfoaluminate hydrates (AFm) with rose-like blossom shapes in the ITZ region of IOTs. When gypsum was insufficient, needle-like ettringite transformed to AFm, which had a positive effect on the specimen strength.

Additionally, a small number of cracks with a width of approximately 0.2 μm were observed on the surface of IOT particles (Figure 9d). The higher the IOT content, the more the cracks in aggregates, resulting in reduced mechanical properties of concrete. Therefore, the content of IOTs needed to be strictly controlled to avoid the loss of mechanical properties. This finding suggested that the microstructure characteristics and mechanical properties were closely related.

The SEM microstructures of TRAC-PP0.6 at 25–1000 magnification are presented in Figure 9e–j. From Figure 9e, it can be seen that fibers are distributed in the matrix in a disordered manner, forming many micro reinforcing ribs. The spherical cavities were released from the cement matrix as a result of fiber pull-out. When the concrete was cracked, the bond between the fiber and cement matrix resulted in fiber yielding (Figure 9f), which also led to the disruption of fiber tip (Figure 9g–i). It was observed that some mortars were attached to the surface of pulled out fiber, and scratch traces were evident on the surface of the fiber (Figure 9j). These findings illustrated that fiber consumed energy during the pull-out process. When load was applied in concrete, the force was transmitted onto the matrix around the fiber by fiber monofilaments, which effectively restrained the generation and propagation of micro-cracks inside the concrete. Figure 9f reflects the ITZ of fiber and cement matrix in concrete. Bending deformation occurred at the fibers during the anti-deformation process under load, and in turn, the fibers embedded within the matrix were debonded with the surrounding matrix. It is worth mentioning that even when macro-cracks occurred, the fibers were still bridging (Figure 9k), thus limiting the propagation of micro-cracks in the concrete. The reduction of cracks in concrete improved the strength and energy absorption capacity of concrete, which explained the mechanism of fiber enhancing the macroscopic mechanical properties of concrete from the micro level.

### 3.5. Freeze–Thaw Resistance

#### 3.5.1. Weight Loss Rate

Freeze–thaw resistance is the ability of concrete to withstand the damage created by the frost heaving of water. That is one of the important indexes to indicate the durability of the concrete, which is usually associated with weight loss rate, relative compressive strength changes, and RDEM as the assessment criteria.

**Figure 10:**
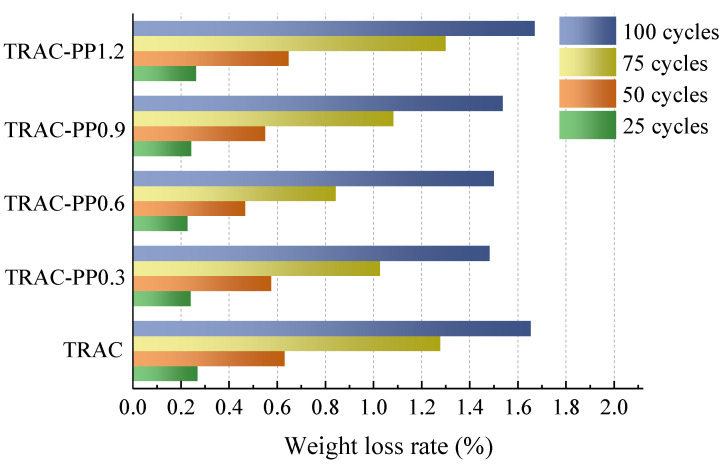
Weight loss rate of all mixtures during freeze–thaw cycles.

The weight loss rate of specimens after freeze–thaw cycles is shown in Figure 10. From the test results, with the increase in freeze–thaw cycles, the weight loss rate for all specimens was increasing. Weight loss caused by freeze–thaw cycles resulted from the swelling of water. Under a freeze–thaw environment, moisture continuously froze, melted, and then repeatedly created an expansion force and penetration pressure to the surrounding matrix. When these pressures exceeded the tensile strength of the surrounding matrix, the concrete will crack. In repeated freezing and thawing, frozen water would again move into the cracks during thawing period, and subsequently, the melted ice refroze. Therefore, as the number of freeze–thaw cycles increased, the width of the original crack gradually widened, the number of micro-cracks gradually increased, and the phenomenon that concrete peeling off intensified.

The weight loss rate was clearly reduced for specimens containing small amounts of PPF compared to specimen containing no PPF. After 25–100 freeze–thaw cycles, the weight loss rate for TRAC was varying from 0.27 to 1.65%, while that of TRAC-PP was ranging from 0.24 to 1.67%. The weight loss rate of TRAC-PP containing 0.3–0.9% PPF was lower than that of TRAC. In this study, TRAC-PP0.6 was optimal in terms of reducing the weight loss rate. For example, after 25, 50, 75, and 100 freeze–thaw cycles, the weight loss rate of TRAC-PP0.6 decreased by 0.04%, 0.16%, 0.43%, and 0.15%, respectively, compared to TRAC.

After a certain amount of PPF was added to the concrete, PPF was intertwined into a fibrous network, which could play a role in supporting aggregates, effectively reducing the weight loss of concrete during the freeze–thaw cycles. Moreover, the crack resistance effect of PPF could reduce the micro-cracks inside concretes caused by the expansion force and penetration pressure. A decrease in the micro-cracks meant a decrease in the seepage channels of frozen water. Thus, the incorporation of small amounts of PPF into TRAC was advantageous for reducing the damage caused by the freeze–thaw process on the cement matrix. After 100 cycles, the weight loss rate of TRAC was 1.65%, while TRAC-PP1.2 showed a higher weight loss rate of 1.67%. This was because cracks tended to propagate along the interface between the cement matrix and aggregates. If the fiber addition reached or even exceeded the critical level, interfacial regions of adjacent fibers overlapped with each other; then, excess weak areas inside the cement matrix could result, thereby leading to a faster failure of concrete.

#### 3.5.2. Relative Compressive Strength

**Figure 11:**
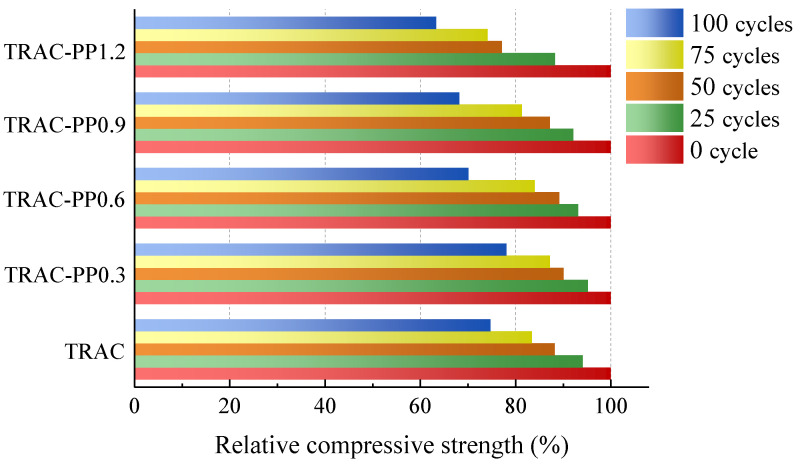
Relative compressive strength of all mixtures during freeze–thaw cycles.

Figure 11 shows the relative compressive strength of specimens after freeze–thaw cycles. It can be seen that as the number of freeze–thaw cycles increased, the relative compressive strength decreased gradually. The reasons are as follows: As the number of cycles increased, the crack expansion increased, the width of cracks increased, and the effective bearing area decreased. A reduction of the effective bearing area further resulted in an increase in the stress concentration near the crack tip. All specimens had higher reduction rate for relative compressive strength between the cycles of 75 and 100 than between 0 and 25. For example, from 0 and 25 cycles, the relative compressive strength of all specimens decreased by 7.46% on average, whereas from 75 and 100 cycles, the relative compressive strength of all specimens decreased by 11.14%. This demonstrated that the deterioration of concrete materials was accelerated with the increasing number of freeze–thaw cycles.

There was no marked difference among the specimens in terms of relative compressive strength when the number of cycles was less than 25. We also observed a slightly increase in the freeze–thaw resistance after a small amount doping of PPF when the freeze–thaw cycle was greater than 25. The freeze–thaw resistance of specimens tended to increase with increasing PPF content for low PPF content (0 to 0.3 wt %), but they decreased for high PPF content (0.6 to 1.2 wt %). After 25, 50, 75, and 100 freeze–thaw cycles, the relative compressive strength of TRAC-PP0.3 increased by 1.1%, 1.9%, 3.8%, and 3.4%, respectively, compared to TRAC. The relative compressive strength was lower than TRAC by 4.7%, 6.5%, and 11.4% in TRAC-PP0.6, TRAC-PP0.9, and TRAC-PP1.2, respectively, after 100 cycles. As it can be seen from the test data, the freeze–thaw resistance of TRAC-PP was even lower than that of TRAC when the PPF content exceeded 0.3%.

The deterioration effects of freeze–thaw cycles on the concretes are mainly due to the disruptive effect of expanding water on the interior structures of concretes. When the specimens were experienced cracking due to the process of repeated freeze–thaw cycles, adding a small amount of PPF had delayed the time of crack propagation. In addition, the slip and fracture behavior of PPF at the crack contributed to consuming energy and improving the mechanical strength when subjected to compression load. Therefore, the relative compressive strength of TRAC was enhanced after PPF doping.

However, excessive PPF resulted in an increase in the weak interfacial zone. Increased interfacial zones would lead to significant declines in intensity. The results implied that the effects of PPF on weight loss rate were much clearer than were those on relative compressive strength.

#### 3.5.3. Relative Dynamic Modulus of Elasticity

**Figure 12:**
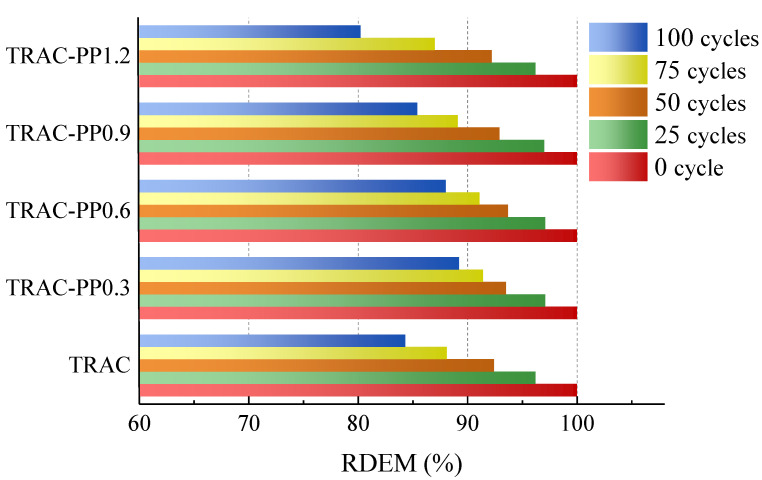
RDEM of all mixtures during freeze–thaw cycles.

The RDEM of specimens after freeze–thaw cycles is shown in Figure 12. The RDEM first increased and then decreased with increasing PPF content. The RDEM values reached a maximum value at the PPF content of 0.3% under the same cycling conditions and cycle numbers, which indirectly suggested that TRAC with a PPF content of 0.3% exhibited the best freeze–thaw resistance. When the content of PPF was increased to 1.2%, the RDEM was significantly lower than that of the control group. This implied that the content of PPF should not be high; otherwise, the freeze–thaw resistance was negatively affected.

The RDEM gap between TRAC-PP0.3, TRAC-PP0.6, and TRAC-PP0.9 was small, and the RDEM of TRAC-PP1.2 was almost the same as TRAC after 25 cycles. After 100 cycles, the RDEM for PPF addition ratios of 0%, 0.3%, 0.6%, 0.9%, and 1.2% was 84.3%, 89.2%, 88%, 85.4%, and 80.2%, respectively, where the RDEM of TRAC-PP1.2 was even 4.1% weaker than that of the control group. The experimental results showed that in terms of freeze–thaw resistance, the TRAC-PP with 0.3–0.6% was considered as the optimum mixture.

## 4. Conclusions

Based on the results analysis above, the following conclusions were drawn:

1. Adding PPF widened the transition area of the stress–strain curves, which was accompanied by improved ductility.
2. As the content of the PPF increased, the peak stress and energy absorption capacity first increased and then decreased, reaching their maximum values at 0.6% content of PPF. However, the effects of PPF on peak stress were not significant. At the same time, it was also found that the peak strains were significantly higher in the TRAC-PP series than in the control group, while the elastic modulus were lower in the TRAC-PP series than in the control group.
3. The stress–strain curves of TRAC-PP were considerably influenced by the content of the PPF. A segmented constitutive model was proposed for the stress–strain curves of TRAC-PP with different PPF content. The calculated curves are in good agreement with the experimental curves.
4. The microstructure of TRAC appeared dense enough. We experimentally found that there were a large amount of C-S-H gel and needle-like ettringite near the IOTs. Meanwhile, a large number of AFm was observed in the ITZ around the IOT particles, and this was known to be beneficial for the macro strength. In addition, a few micro-cracks were found on the IOT surface, which might partially explain the decreased strength as a result of excess use of IOTs.
5. Attached dense hydration products on the PPF surface and the phenomenon of fiber yielding represented fiber would consume energy during the pull-out process. Throughout the cracking process, the fibers transferred the load to the matrix around the fibers by bridging micro-cracks, thus effectively enhancing the strength and energy absorption capacity of concrete. The results of SEM analysis were consistent with those observed in macroscopic mechanical tests.
6. The incorporation of small amounts of PPF into TRAC was advantageous for reducing the damage caused by the freeze–thaw process on the cement matrix. The freeze–thaw resistance first increased and then decreased with increasing PPF content. Additionally, PPF effects were more significant on weight loss rate than relative compressive strength and RDEM. The experimental results demonstrated that the best freeze–thaw resistance was obtained for TRAC-PP with 0.3–0.6% content of PPF. A solid waste utilization rate should be maximized while guaranteeing the mechanical properties and durability. Thus, it was suggested that the content of PPF in TRAC-PP should be 0.6%.

## Figures and Tables

**Table 1:** The performance indicators of NCA, RCA, natural sand, and IOTs.

Aggregate	Apparent Density (kg/m^3^)	Loose Bulk Density (kg/m^3^)	Water Absorption (%)	Crushing Index (%)	Mud Content (%)	Water Content (%)
NCA	2941	1749	1.33	10.3	0.72	0.8
RCA	2436	1467	7	14.8	1.86	3.02
Natural sand	2764	1830	2.12	12	1.2	4.1
IOTs	2745	1824	8.7	19.53	2.9	1.45

**Table 2:** The elemental compositions of natural sand and IOTs.

Component	O	Mg	Al	Si	S	K	Ca	Fe	Ti
Silica sand	60.01	0.71	2.57	32.33	-	0.76	1.56	2.05	0
IOTs	56.49	6.95	8.38	17.46	-	3.02	1.68	5.28	0.74
Cement	47.54	0.74	1.54	6.11	1.10	0.53	56.85	0.93	-

**Table 3:** Main performance indicators of cement.

Water Requirement for Normal Consistency/%	Initial Setting Time/min	Final Setting Time/min	Fineness Modulus (45 μm)	Stability	Flexural Strength (MPa)		Compressive Strength (MPa)	
					3 Days	28 Days	3 Days	28 Days
28	160	280	2.8	Qualified	5.2	6.8	19.5	42.2

**Table 4:** The physical and mechanical characteristic of PPF.

Specific Gravity (kg/cm^3^)	Length (mm)	Diameter (mm)	Tensile Strength (MPa)	Breaking Elongation Rate (%)	Elastic Modulus (MPa)
1.12	22	0.08	>350	12–40	>4000

**Table 5:** Mixture proportions.

Specimen	Water (kg/m^3^)	Ordinary Portland Cement (kg/m^3^)	NCA (kg/m^3^)	RCA (kg/m^3^)	Natural Sand (kg/m^3^)	IOTs (kg/m^3^)	PPF (%)
TRAC	215	537	744	319	400	172	0
TRAC-PP0.3	215	537	744	319	400	172	0.3
TRAC-PP0.6	215	537	744	319	400	172	0.6
TRAC-PP0.9	215	537	744	319	400	172	0.9
TRAC-PP1.2	215	537	744	319	400	172	1.2

**Table 6:** The energy absorption of specimens under compressive test.

Specimens	TRAC	TRAC-PP0.3	TRAC-PP0.6	TRAC-PP0.9	TRAC-PP1.2
	59.02	54.71	83.91	64.67	61.57

## Abbreviations

TRAC	recycled aggregate concrete containing iron ore tailings
PPF	polypropylene fiber
TRAC-PP	fiber-reinforced tailings recycled aggregate concrete
IOTs	iron ore tailings
NCA	natural coarse aggregate
RCA	recycled coarse aggregates
RAC	recycled aggregate concrete
ITZ	interfacial transition zone
SEM	scanning electron microscopy
RDEM	relative dynamic modulus of elasticity
C-S-H	calcium silicate hydrate
AFm	monosulfate calcium sulfoaluminate hydrates
